# The rate of blastocysts production following vitrification with step-wise equilibration of immature mouse oocytes 

**Published:** 2012-09

**Authors:** Reza Mahmoudi, Farzad Rajaei, Iraj Ragardi Kashani, Mehdi Abbasi, Fardin Amidi, Aligholi Sobhani, Iraj Amiri

**Affiliations:** 1 *Cellular and Molecular Research Center, Yasuj University of Medical Sciences, Yasuj, Iran. *; 2 *Department of Anatomy and Infertility Centre, Qazvin University of Medical Sciences, Qazvin, Iran. *; 3 *Department of Anatomy and Embryology, Tehran University of Medical Sciences, Tehran, Iran.*; 4 *Research Center for Endometr and Endometriosis, Hamedan University of Medical Sciences, Hamedan, Iran.*

**Keywords:** *Vitrification*, *Germinal Vesicle stage Oocyte*, *IVM*, *Mouse*

## Abstract

**Background:** Cryopreservation and in vitro maturation (IVM) of oocyte is becoming an important technique in infertility treatment and fertility preservation. Also it has been proposed to establish a genetic resource bank for endangered or commercially important animal species.

**Objective: **The aim of this study was to evaluate viability, maturation and fertilization rate of mouse immature oocytes after single and stepwise vitrification procedure.

**Materials and Methods:** Oocytes were obtained from 4 weeks old female mice 48h after intraperitoneal injection of 7.5 IU pregnant mare serum gonadotropin (PMSG). Collected oocytes before vitrification were exposed to cryoprotectant, which was composed of 30% (v/v) ethylene glycol, 18% (w/v) Ficoll-70, and 0.3 M sucrose, either by single step or in a step-wise way. After vitrification and storage in liquid nitrogen, the oocytes were warmed and washed two times in medium TCM199 and then subjected to IVM, fertilization and subsequent development to blastocysts.

**Results:** The oocytes survival rates after vitrifying-warming (88.96%), maturation rate (73.23%), the capacity of fertilization (57.80%) and embryonic development to blastocyst (16.41%) in the step-wise exposure were significantly higher (p<0.001) compared with corresponding rate in the single step procedure.

**Conclusion:** The results suggest that vitrification with step-wise procedure has positive effects on maturation and developmental capacity of mice germinal vesicle oocytes in compare with single step vitrification procedure.

## Introduction

Cryopreservation and subsequent in vitro maturation (IVM) of immature oocyte is becoming an important and integral part of infertility treatment and fertility preservation (1, 2). Freezing of oocyte could be applied to patients who have severe ovarian diseases such as endometriomas, genital cancer or specific situation such as chemotherapy or radiation (3, 4).

Also because of several advantages IVM, such as low costs, low risk of ovarian hyperstimulation syndrome (OHSS) and simplification of treatment in some infertile couples ,it has been proposed as an alternative for conventional IVF treatment (5, 6). It is established that, slow cryopreservation of mature oocytes is traumatic, partly due to the presence of the temperature-sensitive microtubular spindle which is required for normal fertilization and embryo development (4, 7). 

Vitrification is the process by which water is prevented from forming ice due to the viscosity of a highly concentrated cryoprotectant cooled at an extremely rapid rate. To reduce exposure to the toxic cocktail of cryoprotectants and prevent extreme dehydration, cells are exposed to the cryoprotectants for a very short period to avoid chilling injury and ice crystal formation. However, the spindle in mature oocyte is sensitive to low temperature and in the most of cases disrupts during cryopreservation and it is an important factor that causes low survival rate after freezing of mature oocyte Germinal vesicle (GV)-stage oocytes have not yet formed this spindle and it is an alternative to cryopreservation of oocytes. 

At this stage, chromosomes are decondensed and enclosed within the nuclear membrane and protected from disorders (4-7). Cryopreserved GV-stage human oocytes have been shown to be capable of completing nuclear maturation and becoming fertilized (8, 9). 

During cryopreservation several factors such as exposure time of cells to different cryoprotectant solutions, cryoprotectants concentrations and rate of ice crystal formation affect the survival and viability of oocytes and embryos (11, 12). Also, studies have shown that cumulus cells play an important role on oocyte maturation, since they provide and transfer several known and unknown factors that are essential for normal nuclear and cytoplasmic maturation of oocytes and subsequent embryonic development after fertilization (12, 13). 

GV-stage oocytes which are stripped of cumulus cells have a reduced developmental capacity compared with that of cumulus-enclosed GV-stage oocytes (11, 14, 15). Recently, it is proposed that the cryopreservation of oocyte and embryos using vitrification is superior to slow freezing method (16, 17). Studies have shown that vitrification offers new interesting perspectives in the field of oocyte cryopreservation, demonstrating less traumatic than slow freezing (17, 18). Because vitrification is a method that needs relatively high concentration of cryoprotectants, a step-wise addition of cryoprotectants may reduce the toxic effects of cryoprotectants and is considered to minimize damage due to extreme cell-volume expansion.

The aim of present study was to compare the effect of stepwise and single step exposure to cryoprotectant on the developmental ability of vitrified mouse immature oocyte in an ethylene glycol-sucrose based vitrification media. The oocytes were evaluated by post warming survival, IVM, in vitro fertilization and developmental capacity to blastocyst stage.

## Materials and methods

**Chemical reagents**

All chemicals were purchased from Sigma Chemical Co., St. Louis, MO, except for the ones specially described. This study was carried out as experimental research on mouse GV oocytes. GV oocytes were obtained from 3-4 week old ICR strain female mice (Japan SLC Inc., Shizuoka, Japan)) the animals were kept under controlled condition (12 hr light: 12 hr dark). 

Mice were stimulated by an intraperitoneal injection of 7.5 IU PMSG (Teikokuzouki, Tokyo, Japan). 48h later the animals were killed by cervical dislocation and the ovaries removed in Hepes-buffered human tubal fluid medium (HTF) supplemented with 5mg/ml BSA. The GV-stage oocytes of ovarian antral follicles were released by puncturing with a 28G micro-injection needle under a stereomicroscop. The GV cumulus oocyte complex (GV-COC) was randomly allocated to three groups including control, stepwise and single-step vitrification group.

**Oocytes vitrification**

Vitrification of oocytes was done according to the method described by Kasai *et al* (19). In the stepwise group, the COCs were exposed for 5 min to 200 ml droplets of solution A, which was composed of 10% (v/v) ethylene glycol, 4.5% (W/V) Ficoll-70, and 0.075 M sucrose, then for 2 min to 200 μl drop of solution B, which was composed of 20% (V/V) ethylene glycol, 9.0% (W/V) Ficoll-70, and 0.15 M sucrose, and finally for 1 min to 200 μl drop of solution C, which was composed of 30% (V/V) ethylene glycol, 18% (W/V) Ficoll-70, and 0.3 M sucrose in 4 well dishes. In the single step group, the COCs were exposed for 1 min to 200 μl drop of solution C.

The procedures were performed at room temperature of 22-24^o^C. After equilibrium, 10-15 GV oocytes were loaded into a 0.25 ml plastic straw (IVM, I Aigle, France). The straw was filled with 1 cm of vitrification solution, 0.5 cm of air, 2 cm of vitrification solution containing oocytes, 0.5 cm of air, and 3.5 cm of vitrification solution. The straw then was plunged into liquid nitrogen. After storage for 1-5 days, the straw was taken out of the liquid nitrogen, held in the air for 10 s, and then plunged into water of 37^o^C for 10 s. 

The straw was removed from water and wiped dry. It was cut with scissors and the contents containing oocytes were expelled into 400-μl drop with a sequential series of 0.5, 0.25, and 0.125 M sucrose by keeping for 90 s in each solution, and washed for 6 min in TCM199 medium supplemented with 20% FBS. 

**Maturation of GV oocytes**

The vitrified-thawed GV oocytes or fresh GV oocytes (control group) were cultured in IVM medium (α-MEM). 16-18 h after culture, the GV-COC oocytes with first polar body were defined as mature MII oocytes.

**In vitro fertilization and development**

Spermatozoa from male mice of 12 weeks old were released by cauda Epididymis puncture in to IVF medium TYH medium supplemented with 4 mg/ml BSA. The sperm were suspended in 200μl droplet of the IVF medium, Were covered with the mineral oil and were incubated at 37ºC for 1-2 h in a humidified atmosphere of 5% CO_2_. For capacitation mature oocyte (n=15-20) were placed in separate 200μl droplet of IVF Medium under mineral oil. 

Sperm mixture (10-20μl) was add to each droplet to obtain a concentration of 1-2×10^6^ motile sperm/ml. after co-incubation for 5 h at 37^o^C, the oocytes were removed and washed in fresh TYH medium and placed in a 5% CO_2_ incubator at 37ºC. At 6-8 h post-insemination, embryos with two distinct pronuclei and the second polar body was classified as PN stage observed under a phase-contrast inverted microscope. The pronuclei stage transferred to 100μl of KSOM (potassium Simplex optimized medium) under mineral oil. They were assessed for cleavage to the 2-cell stage 24 hours and for blastocyste stage 120 hours after fertilization.

**Statistical analysis**

Collected data were analyzed by Chi-Squar test. The difference in values of survival, maturation, fertilization and developmental rate, were considered significant when p<0.05.

## Results

**Survival and IVM of vitrified GV oocytes**

The survival and maturation rates in the stepwise group were significantly higher than single step but maturation rate in control group was significantly higher than both of them (p<0.001) (Table I).

**In vitro fertilization and developmental of vitrified GV**

**Figure 1:**
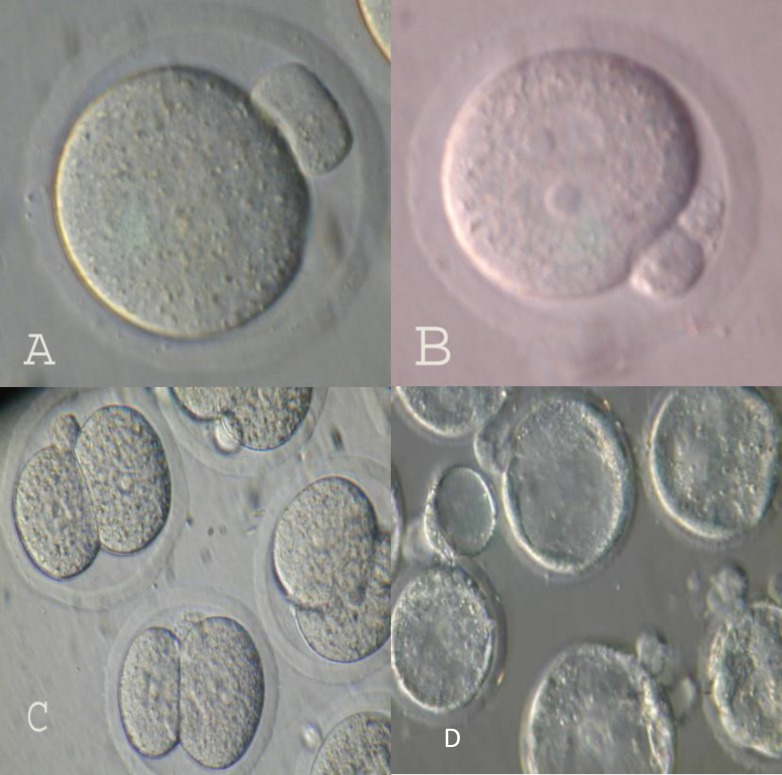
Maturation and fertilization and development to 2-cell stage of GV oocytes after vitrification. A) Maturation to metaphase II 24h after culture in maturation medium. B) Fertilization oocyte (2PN) after 6-8h after insemination. C) Development to 2-cell stage 24h and development to blastocysts 120h after insemination

The rates of fertilization and embryonic development to blastocyst stage in the control group were significantly higher than both of the vitrified groups. Among the vitrified groups the fertilization and developmental rates in the stepwise group were significantly higher than single step group(fig.1)and (tableII) (p<0.001). 

**Table I:** Survival and IVM rates of vitrified mouse GV oocyte

**Group**	**No. of GV oocyte examination**	**Oocyte survival (%)**	**Final stage of oocytes maturation**		
			**No of GV (%)**	**No of GVBD (%)**	**Maturation MII (%)**
Control	252	252 (100)^a^	0	24 (9.47)	226 (89.41)^a^
Stepwise	150	134 (88.96)^b^	2 (1.34)	34 (25.37)	98 (73.23)^b^
Single step	168	116 (70.6)	9 (6.89)	35 (30.67)	72 (62.42)

a-b Values with different superscript within same column are significantly different (p<0.001).

**Table II:** Fertilization, cleavage rates and blastocyst formation rates of vitrified mouse GV oocytes

**Group**	**Fertilization (%)**	**2 cell (%)**	**4 cell (%)**	**8 cell (%)**	**Morula (%)**	**Blastocyst (%)**
Control	204 (80.76)^a^	192 (75.58)^a^	177 (69.38)^a^	157 (61.58)^a^	139 (54.65)^a^	117 (45.62)^a^
Stepwise	77 (57.80 )^b^	70 (53.19 )^b^	50 (36.50 )^b^	39 (27.35 )^b^	32 (22.20 )^b^	22 (16.41 )^b^
Single step	53 (45.68 )	44 (38.95 )	32 (28.45 )	22 (18.76 )	16 (13.47 )	6 (4.84 )

a-b Values with different superscript within same column are significantly different (p<0.001).

## Discussion

The cryopreservation of human immature oocytes has distinct advantages compared to mature oocytes. Immature oocytes may circumvent the problem of spindle damage that frequently occurs in mature oocytes during cryopreservation, since the chromosomes remain within the nucleus. This unique position protects them from direct exposure to low temperatures and cryoprotectants (19). 

In this study, we showed that cryopreservation by vitrification enabled mouse GV stage oocytes to survive, mature, fertilize and develop to blastocyst. However, successful cryopreservation of mouse GV oocytes has been reported in previous studies (20-24). But the fertilization and cleavage rates are still poor, probably due to high toxic effects of cryoprotectants, following vitrification and in vitro maturation of immature oocytes (25). 

Since vitrification is a nonequilibrium cryopreservation method that needs a relatively high concentration of cryoprotectants, a step-wise addition of cryoprotectants may reduce the toxic effects of cryoprotectants and be considered to minimize damage due to extreme cell-volume expansion. In compare to previous study our results show higher maturation fertilization and blastocyst formation rate. For example Hochi *et al* in 1998 vitrified immature bovine oocytes in straws by using a mixture of 40% EG, ficoll and sucrose as a vitrification medium (24). 

They reported 47.5% fertilization rate from the vitrified bovine oocytes. In another study, in which immature bovine oocytes were vitrified using a mixture 2.5M EG, ficoll and sucrose in (open pulled straws) OPS (26), a successful maturation rate of 60% was recorded. Cetin *et al* in 2006 vitrified immature bovine oocytes, 34.1% of oocytes reached the MII stage in EG group (27). In the present study the lower maturation rate of in the single step with EFS30 indicating that, during vitrification of mouse GV-COCs, the process of gradual equilibration seems to adjust permeability of plasma membrane, which may contribute to maintaining the oocyte and cumulus cells, and/or may decrease rapid changes in osmotic pressure. 

On the other hand, similar to our results, Abe *et al* in 2005 showed that survival, fertilization, maturation and developmental rates of bovine GV-COCs in stepwise vitrification were significantly higher than those vitrified in single-step (21). Also Anon *et al* in 2003 and 2005 reported higher survival, maturation and developmental rate to blastocysts using ultrarapid vitrivication accompanied with step-wise equilibration in mouse GV oocytes than single step vitrified group (28, 29). 

The findings of the present study show that the modification of Kasai vitrification method in a stepwise manner exposure of mice GV oocytes to cryoprotectants during equilibration phase of vitrification, can result in better maturation, fertilization and developmental rates. Recently, Kuwayama introduced a Highly efficient vitrification method for cryopreservation of human oocytes and embryos using cryotop (30). However the method of Kuwayam is useful for human embryo and oocyte but is not easily applicable in studies on mouse, because using of inexpensive equipment is very important in the studies on mouse and cryotop is very expensive. 

One of the major advantages of our method in comparison with Kuwayama is applying conventional cryopreservation straws instead of cryotop. Therefore in terms of economic; our method is more cost effective and applicable in the basic research. Our method allows researchers to vitrify mouse immature oocyte in a simpler and cheaper manner.

In conclusion, the current results demonstrated that vitrification of murine GV oocytes accompanied with step-wise equilibration using conventional straws showed improved results for viability and production of blastocysts, suggesting that this method may be a easy, useful and low cost strategy for the cryopreservation of immature oocytes.

## References

[1] Atabay EC, Takahashi Y, Katagiri S, Nagano M, Koga A, Kanai Y (2004). Vitrification of bovine oocytes and its application to intergeneric somatic cell nucleus transfer. Theriogenology.

[2] Amorim CA, Goncàlves PB, Figueiredo JR (2003). Cryopreservation of oocytes from pre-antral follicles. Hum Rep Update.

[3] Paynter SJ (2000). Current status of the cryopreservation of human unfertilized oocytes. Hum Reprod Update.

[4] Mahmoudi R, Amiri I, Pasbakhsh P, Ragardi Kashani I, Abbasi M, Farid Aboulhasani F (2008). The effects of vitrification on spindle apparatus of invitro matured germinal vesicle in mice. Iran J Reprod Med.

[5] Trounson AO, Wood C, Kausche A (1994). IVM and the fertilization and developmental competence of oocytes recovered from untreated polycystic ovarian patients. Fertil Steril.

[6] Wu J, Zhang L, Wang X (2001). IVM, fertilization and embryo development after ultra-rapid freezing of immature human oocytes. Reproduction.

[7] Cao Y, Xing Q, Zhang ZG, Wei ZL, Zhou P, Cong L (2009). Cryopreservation of immature and in-vitro matured human oocytes by vitrification. Reprod Biomed Online.

[8] Son WY, Park SE, Lee KA, Lee WS, Ko JJ, Yoon TK (1996). Effects of 1,2-propanediol and freezing-thawing on the in vitro developmental capacity of human immature oocytes. Fertil Steril.

[9] Schroeder AC, Eppig JJ (1984). The developmental capacity of mouse oocytes that matured spontaneously in vitro is normal. Dev Biol.

[10] Mahmoudi R, Sobhani A, Pasbakhsh P, Abolhasani F, Amiri I, Salehnia M (2005). The Effects of cumulus cells on IVM of mouse germinal vesicle stage oocytes. Iran J Reprod Med.

[11] Mahmodi R, Abbasi M, Amiri I, Ragardi Kashani I, Pasbakhsh P, Saadipour Kh (2009). Cumulus cell role on mouse germinal vesicle oocyte maturation, fertilization, and subsequent embryo development to blastocyst stage in vitro. Cell Journal.

[12] Nagai T (2001). The improvement of IVM systems for bovine and porcine oocytes. Theriogenology.

[13] Khosravi-Farsani S, Sobhani A, Amidi F, Mahmoudi R (2010). Mouse oocyte vitrification: the effects of two methods on maturing germinal vesicle breakdown oocytes. J Assist Reprod Genet.

[14] Anderiesz C, Trounson AO (1995). The effect of testosterone on the maturation and developmental capacity of murine oocytes in vitro. Hum Reprod.

[15] Chang M (1955). The maturation of rabbit oocytes in culture and their maturation, activation, fertilization, and subsequent development in the fallopian tubes. J Exp Zool.

[16] Hashimoto S, Saeki K, Nagao Y, Minami N, Yamada M, Utsumi K (1998). Effects of cumulus cell density during IVM of the developmental competence of bovine oocytes. Theriogenology.

[17] Goud PT, Goud AP, Qian C, Laverge H, Van der Elst J, De Sutter P (1998). In-vitro maturation of human germinal vesicle stage oocytes: role of cumulus cells and epiderminal growth factor in the culture medium. Hum Reprod.

[18] Anderiesz C, Ferraretti A, Magli C, Fiorentino A, Fortini D, Gianaroli L (2000). Effect of recombinant human gonadotropins on human, bovine and murine oocyte meiosis, fertilization and embryonic development in vitro. Hum Reprod.

[19] Zhiguo Z, Yu Liu, Qiong X, Ping Z, Yunxia C (2011). Cryopreservation of human failed-matured oocytes followed by in vitro maturation: vitrification is superior to the slow freezing method. Reprod Biol Endocrinol.

[20] Kassai M, Komi JH, Takakamo A, Tsudera H, Sakurai T (1999). Asimple method for mouse embryo cryopreservation in low toxicity vitrification solution, without appreciable loss of viability. J Reprod Fertil.

[21] Abe Y, Hara K, Matsumoto H, Kobayashi J, Sasada H, Ekwall H (2005). Feasibility of a nylon-mesh holder for vitrification of bovine germinal vesicle oocytes in subsequent production of viable blastocysts. Biol Reprod.

[22] Cooper A, Paynter SJ, Fuller BJ, Shaw RW (1998). Differential effects of cryopreservation on nuclear or cytoplasmic maturation in vitro in immature mouse oocytes from stimulated ovaries. Hum Reprod.

[23] Wongsrikeao P, Kaneshige Y, Ooki R, Taniguchi M, Agung B, Nii M (2005). Effect of the removal of cumulus cell on the nuclear maturation, fertilization and development of porcine oocytes. Reprod Dom Amin.

[24] Hochi S, Ito K, Hirabayashi M, Ueda M, Kimura K, Hanada A (1998). Effect of nuclear stages during IVM on the survival of vitirified-warmed bovine oocytes. Theriogenology.

[25] Saragusty J, Arav A (2011). Current progress in oocyte and embryo cryopreservation by slow freezing and vitrification. Reproduction.

[26] Hurt AE, Landim F, Seidel GE, Squires EL (2000). Vitrification of immature and mature equine and bovine oocytes in an ethylene glycol, ficoll and sucrose solution using open-pulled straws. Theriogenology.

[27] Cetin Y, Bastan A (2006). Cryopreservation of immature bovine oocytes by vitrification in straws. Anim Reprod Sci.

[28] Aono N, Naganuma T, Abe Y, Hara K, Sasada H, Sato E (2003). Successful production of blastocysts following ultrarapid vitrification with step-wise equilibriation of germinal vesicle-stage mouse oocytes. J Reprod Dev.

[29] Aono N, Abe Y, Hara K, Sasada H, Sato E, Yoshida H (2005). Production of live offspring from mouse germinal vesicle-stage oocytes vitrified by a modified stepwise method, SWEID. Fertil Steril.

[30] Kuwayama M (2007). Highly efficient vitrification for cryopreservation of human oocytes and embryos: the Cryotop method. Theriogenology.

